# Acrylamide induces HepG2 cell proliferation through upregulation of miR-21 expression

**DOI:** 10.7555/JBR.31.20170016

**Published:** 2017-05-27

**Authors:** Yuyu Xu, Pengqi Wang, Chaoqi Xu, Xiaoyun Shan, Qing Feng

**Affiliations:** 1. Department of Nutrition and Food Safety, School of Public Health, Nanjing Medical University, Nanjing, Jiangsu 211166, China; 2. Station of Sanitary Surveillance of Lianyungang, Lianyungang, Jiangsu 222002, China; 3. University of South China, Hengyang, Hunan 421000, China.

**Keywords:** acrylamide, proliferation, miR-21

## Abstract

Acrylamide, a potential carcinogen, exists in carbohydrate-rich foods cooked at a high temperature. It has been reported that acrylamide can cause DNA damage and cytotoxicity. The present study aimed to investigate the potential mechanism of human hepatocarcinoma HepG2 cell proliferation induced by acrylamide and to explore the antagonistic effects of a natural polyphenol curcumin against acrylamide *via* miR-21. The results indicated that acrylamide (≤100 μmol/L) significantly increased HepG2 cell proliferation and miR-21 expression. In addition, acrylamide reduced the PTEN expression in protein level, while induced the expressions of p-AKT, EGFR and cyclin D1. The PI3K/AKT inhibitor decreased p-AKT protein expression and inhibited the proliferation of HepG2 cells. In addition, curcumin effectively reduced acrylamide-induced HepG2 cell proliferation and induced apoptosis through the expression of miR-21. In conclusion, the results showed that acrylamide increased HepG2 cell proliferation *via* upregulating miR-21 expression, which may be a new target for the treatment and prevention of cancer.

## Introduction

Acrylamide, an essential chemical, has been used to synthesize various polymers which have been applied in many fields, such as synthetic dye industry, waste water treatment, cosmetic manufacture, paper production and experimental researches^[1]^. Since 2002, people began to pay attention to the hazard of acrylamide because it was detected in carbohydrate-rich foods cooked at a high temperature and its concentration was 500 times more than the maximum acceptable level in water^[2]^. Exposure to acrylamide may occur *via* skin contact, dust inhalation, water pollution, tobacco smoke and common foods^[3]^. Acrylamide can be spread to all our organs and tissues. Approximately 6% of urinary excretion of acrylamide is converted to more toxic compound glycidamide by cytochrome P450 2E1 (CYP2E1)^[4]^. Additionally, both acrylamide and glycidamide can react with proteins and DNA to form adducts that cause protein and DNA damage^[5]^. Acrylamide has been classified as probably carcinogenic to humans by the International Agency for Research on Cancer (IARC) in 1994. In recent years, a number of animal studies have shown that acrylamide has neurotoxicity and tumorigenicity^[6^–^7]^.

MicroRNAs (miRNAs) are a class of endogenous noncoding RNA with 18-22 nucleotides^[8]^. MiRNAs play critical roles in various biological processes, including proliferation, differentiation, apoptosis, control of developmental timing, and multiple human cancers through silencing and cleaving the target mRNAs *via* binding to their 3′-untranslated region (3′-UTR)^[9^–^11]^. A number of studies have reported that overexpression of miR-21 is present in various types of cancer including hepatocellular cancer^[12]^. Moreover, studies have showed that miR-21 is associated with cell proliferation, migration and invasion in malignant hepatocytes, which indicates that miR-21 may conduce to tumor metastasis^[12^–^13]^. Besides, various prediction algorithms are employed to identify the potential targets of miR-21, including phosphatase and tensin homolog (PTEN) and tyrosine phosphatase non-receptor type 14 (PTPN14). PTEN as a dual lipid and protein phosphatase, has been verified as a tumor suppressor in many types of cancer^[14^–^15]^. PTEN can function as a potent negative regulator of the PI3K/AKT signaling pathway, which can inhibit cell proliferation and induce apoptosis^[16^–^18]^. Overexpression of miR-21 can decrease the level of PTEN and promote tumor cell proliferation, migration and invasion^[12]^. Currently, no study has been conducted to determine whether miR-21 expression is altered by acrylamide in human hepatoma cells.

Our previous study found that the natural polyphenol curcumin and green tea polyphenol epigallocatechin-3-gallate (EGCG) significantly reduce HepG2 cell proliferation^[19]^. In the current study, we showed that curcumin reversed acrylamide-induced HepG2 cell proliferation and promoted cell apoptosis through downregulation of miR-21.

## Materials and methods

### Cell culture and reagents

HepG2 cells were purchased from Cell Center of Chinese Academy of Medical Sciences (Beijing, China). The cells were cultured in Dulbecco's Modified Eagle Medium (DMEM, Gibco, Carlsbad, CA, USA) supplemented with 10% fetal bovine serum (Gibco, Grand Island, CA, USA) and 1% penicillin-streptomycin mixed solution (Beyotime, Shanghai, China). Cells were maintained at 37°C in a humidified 5% CO_2_ atmosphere. Acrylamide (purity>99.5%, dissolved in ddH_2_O in a concentration of 100 mmol/L), curcumin (purity>80%, dissolved in DMSO in a stock concentration of 50 mmol/L), LY294002, the inhibitor of PI3K/AKT were purchased from Sigma-Aldrich (St. Louis, MO, USA).

### MTT assay

Cells were seeded at a density of 4,000 per well in a 96-well plate with 180 µL medium and incubated overnight at 37°C, 5% CO_2_. After the indicated treatment, cells were incubated with methyl thiazol tetrazolium bromide (MTT, Amresco, OH, USA) solution (5 mg/mL) at 37°C, 5% CO_2_ for 4 hours. Then DMSO was added to each well to dissolve formed formazan crystals at room temperature for 10 minutes. Subsequently the solution was read in a microplate reader (Tecan Infinite M200, Mannedorf, Switzerland) at 490 nm.

### Western blot analysis

After the indicated treatments, total cell extracts were obtained and lysed by RIPA buffer (KeyGENBioTECH, Nanjing, China). Protein concentrations were determined according to BCA Protein Assay Kit (Beyotime, Shanghai, China). The extracted proteins in the cell lysates were isolated by sodium dodecyl sulfate-polyacrylamide gel electrophoresis (SDS-PAGE) and transferred to polyvinylidene difluoride membranes (Millipore, Billerica, MA, USA). The primary antibodies were as follows: rabbit anti-EGFR monoclonal antibody (Cell Signaling technology, Danvers, MA, USA), anti-PTEN monoclonal antibody (Cell Signaling technology, Danvers, MA, USA), anti-p-AKT(Ser473) monoclonal antibody (Cell Signaling technology, Danvers, MA, USA), anti-AKT monoclonal antibody (Cell Signaling technology, Danvers, MA, USA), anti-cyclin D1 monoclonal antibody (Santa Cruz Biotechnology, Dallas, USA), anti-Bcl2 monoclonal antibody (Santa Cruz Biotechnology, Dallas, USA), anti-Bax monoclonal antibody (Santa Cruz Biotechnology, Dallas, USA), anti-CYP2E1 monoclonal antibody (Epitomics, CA, USA) and mouse anti-β-actin monoclonal antibody (BOSTER, Wuhan, China). Secondary antibodies include HRP-Conjugated AffiniPure Goat Anti-rabbit IgG (ZSGB-BIO, Beijing, China). Immunoreactive proteins were visualized using ECL western blotting detection regents (GE Health-care, Buckinghamshire, UK).

### EdU fluorescence staining

The HepG2 cells were seeded at a density of 4,000 per well in a 96-well plate. After the indicated treatment, the 5-ethynyl-2'-deoxyuridine (EdU) fluorescence staining was applied to determine the newly-synthesized DNA according to the manufacturer's protocol of Cell-Light^TM^EdU DNA Cell Proliferation Kit (RiboBio, Guangzhou, China).

### Transfection of siRNA, miRNA mimics and inhibitors

Cells were incubated at a density of 2.5 × 10^5^ per well in a six-well plate. After the indicated treatment, HepG2 cells were transfected with pre-designed human CYP2E1 siRNA and siRNA control (RiboBio, Guangzhou, China) using Lipofectamine 2000 reagent (Invitrogen, Carlsbad, Calif, USA). The primer sequences were as follows: CYP2E1 siRNA (Forward: 5′-ATGTCTGCCTCGGAGTGA -3′, Reverse: 5′-GGAAGAGGTTCCCGATGATG-3′). The mimics and inhibitors of the hsa-miR-21were purchased from RiboBio (Guangzhou, China), which were transfected into the cells using Lipofectamine 2000 reagent (Invitrogen, Carlsbad, Calif, USA). All steps were performed following the manufacturer's protocol.

### Real-time polymerase chain reaction

Total RNA was isolated from cells using the RNAiso Plus (TakaRa Bio Technology, Dalian, China) according to the manufacturer's protocol. Reverse transcription was performed using the PrimeScript^TM^RT Master Mix (TakaRaBio Technology, Dalian, China) and the cDNA fragments were analyzed by SYBR® Premix EX Taq^TM^II (TakaRaBio Technology, Dalian, China) with the Applied Biosystems, 7300 Real Time PCR System (Applied Biosystems, Foster City, CA) according to the manufacturer's instructions. The primer sequences were as follows: hsa-miR-21 (5′-uagcuuaucagacugauguuga-3′). The relative expression of miRNA was analyzed by the Eq. (2)^-DDCt^ and normalization with U6.

### Flow cytometry

HepG2 cells were plated at a density of 5 × 10^6^/dish and apoptosis was detected by flow cytometry analysis using Annexin V-FITC and propidium iodide (PI) staining assay (KeyGENBioTECH, Nanjing, China) after the indicated treatment. All steps were carried out following the manufacturer's instructions.

### Colony formation assay

After the indicated treatment, HepG2 cells were seeded (200/well) in a 6-well plate and the medium was replaced every 3 days. An aggregate consisting of 50 or more cells was defined as a colony. Two weeks later, cells were fixed and stained with crystal violet staining solution (Beyotime, Shanghai, China).

### Hoechst 33258 staining

After the HepG2 cells were seeded in a 6-well plate and incubated for 24 hours, curcumin was added at the indicated concentrations and incubated for 24 hours. Then the cells were fixed in 4% paraformaldehyde for 15 minutes, and washed twice with PBS for 5 minutes. The cells were washed twice with PBS again after being stained with 500 µl Hoechst 33258 (Beyotime, Shanghai, China) for 5 minutes. The stained nuclei were observed under an inverted fluorescence microscope (Olympus, Tokyo, Japan).

### Statistical analysis

Data were presented as the mean±standard deviation (SD) of at least three independent experiments. Western blotting was quantified using Image J, and all the proteins detected by Western blotting were calculated normalization with β-actin. Statistical significance of differences between two or more groups was analyzed by student's two-tailed *t*-test or one-way analysis of variance (ANOVA). Statistical significance was set at *P<0.05 and **P<0.01.

## Results

### Acrylamide induced the expression of miR-21 in HepG2 cells

**Fig.1:**
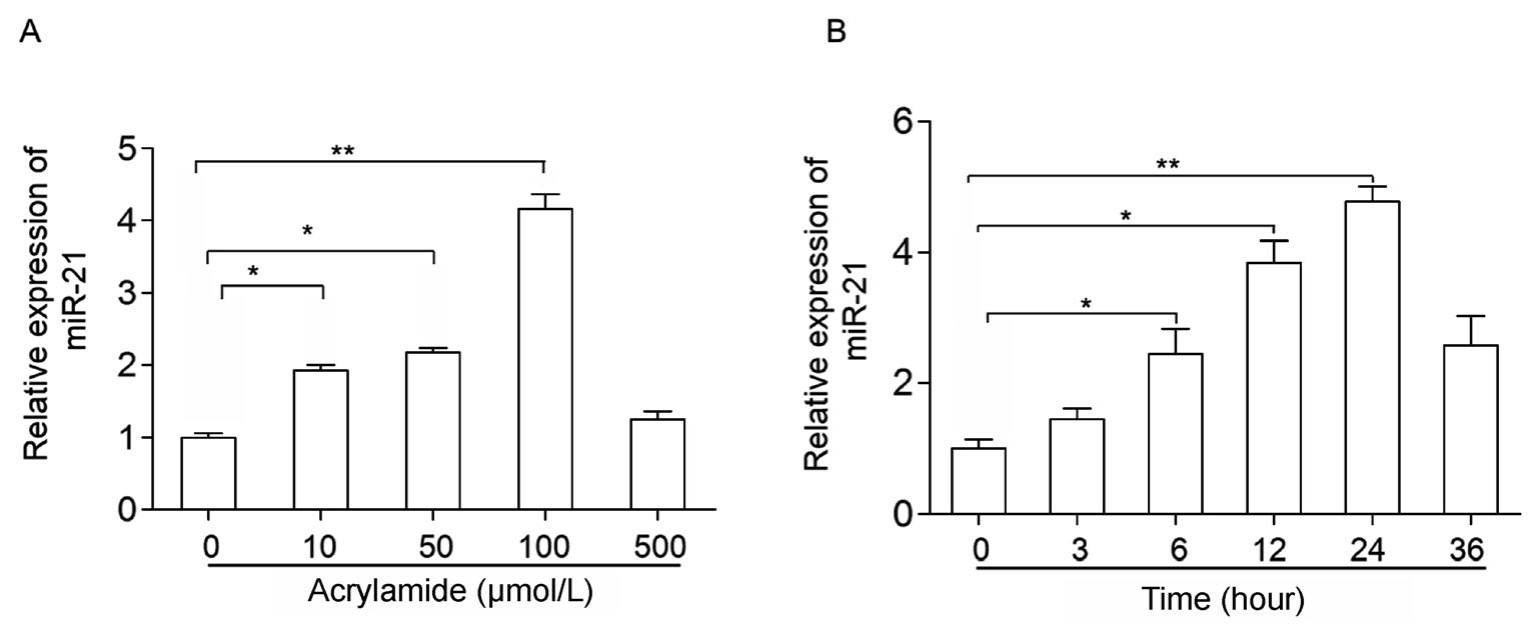
Acrylamide induced miR-21 expression in HepG2 cells. A: Level of miR-21 after treatment with acrylamide (0 ¦̭ol/L, 50 ¦̭ol/L, 100 ¦̭ol/L, 500 ¦̭ol/L) for 24 hours, as analyzed by qRT-PCR. B: Level of miR-21 after treatment with acrylamide at 100 ¦̭ol/L for the indicated time points (0, 3, 6, 12, 24, and 36 hours), as tested by qRT-PCR. *P < 0.05, **P < 0.01.

To investigate whether miR-21 participates in HepG2 cell proliferation induced by acrylamide, the cells were treated with the indicated concentrations of acrylamide for 24 hours. Total RNA was isolated from acrylamide-treated HepG2 cells and quantified by qRT-PCR. The results showed that acrylamide (≤100 µmol/L) induced the expression of miR-21 in a dose-dependent manner. It presented a 4-fold rise when the concentration was at 100 µmol/L (*Fig. 1A*). Then 100 µmol/L acrylamide was chosen to treat HepG2 cells for the indicated time periods because the most significantly enhanced expression of miR-21 occurred at this dose. Results revealed that 100 µmol/L acrylamide promoted the expression of miR-21 in a time-dependent manner in HepG2 cells, and the expression of miR-21 presented a 4.5-fold rise when the cells were treated for 24 hours (*Fig. 1B*).

### Acrylamide downregulated the expression of PTEN while upregulating the expression of p-AKT in HepG2 cells

**Fig.2:**
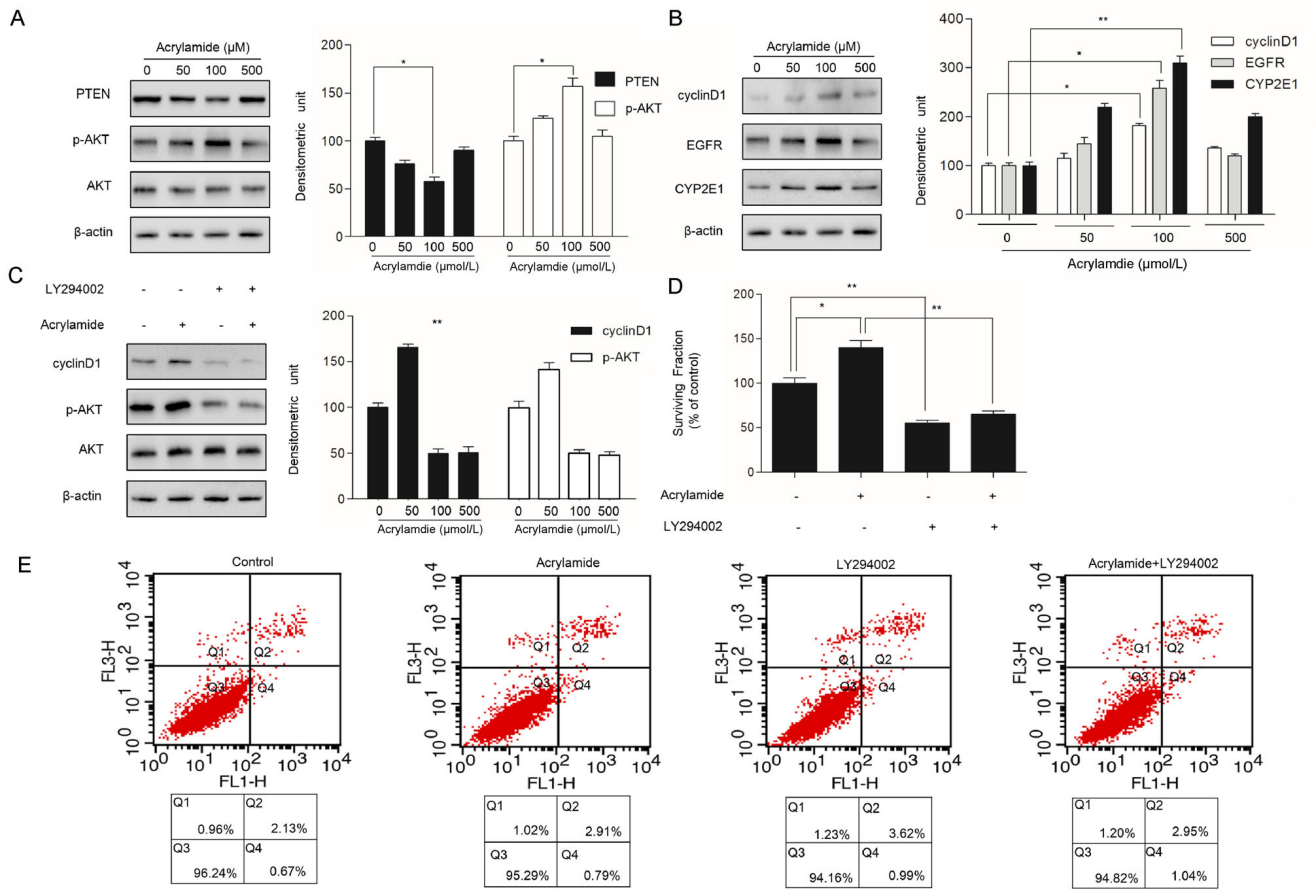
Acrylamide downregulated the expression of PTEN and upregulated the expression of p-AKT in HepG2 cells. A and B: The expression of PTEN, p-AKT, cyclin1, EGFR and CYP2E1 after treatment with acrylamide (0 ¦̭ol/L, 50 ¦̭ol/L, 100 ¦̭ol/L, 500 ¦̭ol/L) for 24 hours. C: The expression of cyclin D1, p-AKTafter treatment with acrylamide (100 ¦̭ol/L), LY294002 (20 ¦̭ol/L), and the combination. A, B and C, as tested by Western blotting. D and E: The surviving fraction and apoptosis rate of HepG2 cells after treatment with acrylamide (100 ¦̭ol/L), LY294002 (20 ¦̭ol/L), and the combination, as measured by MTT assay and flow cytometry, respectively. *P < 0.05, **P < 0.01.

To explore whether acrylamide alters the level of PTEN, AKT and cyclin D1 protein expression, HepG2 cells were treated with the indicated acrylamide for 24 hours. Several protein expressions were analyzed by Western blotting assays. The results indicated that acrylamide, especially at 100 µmol/L, restrained PTEN expression while activating the level of p-AKT and upregulating the level of cyclin D1(*Fig. 2A* and *B*). Our previous studies showed that not only proliferation-related factors, EGFR, cyclin D1 and CYP2E1 expression were upregulated, but also NF-κB was activated in the process of acrylamide promoting HepG2 proliferation^[19]^. To further explore AKT expression in acrylamide inducing HepG2 cell proliferation, LY294002, an inhibitor of PI3K/AKT, was employed to suppress the phosphorylation of AKT after the indicated treatment. p-AKT expression as well as cyclin D1 expression was downregulated by LY294002 (*Fig. 2C*). The results indicated that cell proliferation induced by acrylamide was suppressed by LY294002 (*Fig. 2D*). Besides, in order to distinguish the contribution of proliferation inhibition and apoptosis induction to cell viability, cell viability was evaluated by annexin-V/PI staining (*Fig. 2E*), and there was no statistical significance between the treatment groups, which showed that LY294002 can inhibit HepG2 cell proliferation rather than inducing HepG2 cell apoptosis.

### miR-21 inhibitor reversed HepG2 cell proliferation induced by acrylamide

**Fig.3:**
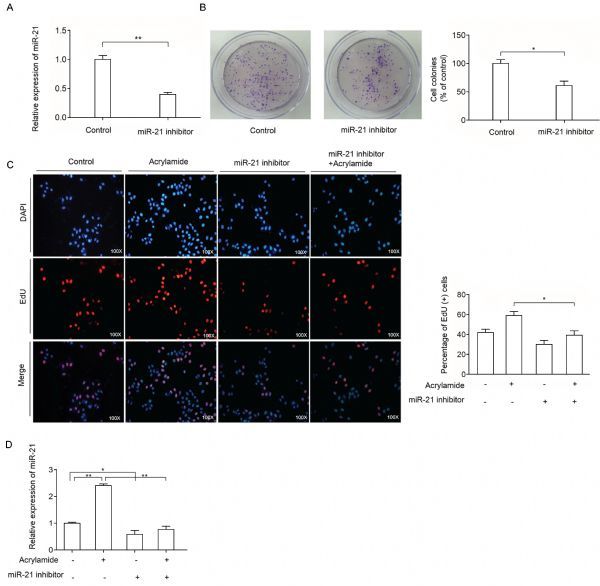
MiR-21 inhibitor reversed HepG2 cell proliferation induced by acrylamide. A and B: Level of miR-21 and the rate of colony formation after the cells were transfected with miR-21 inhibitor (100 nmol/L), as analyzed by qRT-PCR and colony formation assay. C: HepG2 cell proliferation after treatment with acrylamide (100 ¦̭ol/L), miR-21 inhibitor (100 nmol/L) and the combination, as analyzed by EdU staining assay. D: The level of miR- 21 after treatment with miR-21 inhibitor (100 nmol/L) and acrylamide (100 ¦̭ol/L) in HepG2 cells. *P < 0.05, **P < 0.01.

To examine the effect of miR-21 on HepG2 cell proliferation, miR-21 inhibitor was transfected to inhibit the expression of miR-21 in HepG2 cells. As shown in *Fig. 3A*, miR-21 level in the treatment group decreased to 40% compared to the control group after the indicated treatment. Then, the effect of combination of acrylamide and miR-21 inhibitor on HepG2 cell proliferation was examined. As shown in *Fig. 3B*, miR-21 inhibitor reduced colony growth, which indicated that miR-21 inhibitor reversed HepG2 cell proliferation. Moreover, EdU staining was performed to further testify the effect of miR-21 on HepG2 cell proliferation induced by acrylamide. The results indicated that miR-21 inhibitor can reverse HepG2 cell proliferation caused by acrylamide, as well as suppress the HepG2 cell proliferation (*Fig. 3C*). In *Fig. 3D*, the level of miR-21 has slightly changed in the combination of miR-21 inhibitor and acrylamide group. All the results demonstrated that miR-21 was essential in HepG2 cell proliferation caused by acrylamide.

### miR-21 regulated the signaling pathway of miR-21/ PTEN/ AKT in HepG2 cells

**Fig.4:**
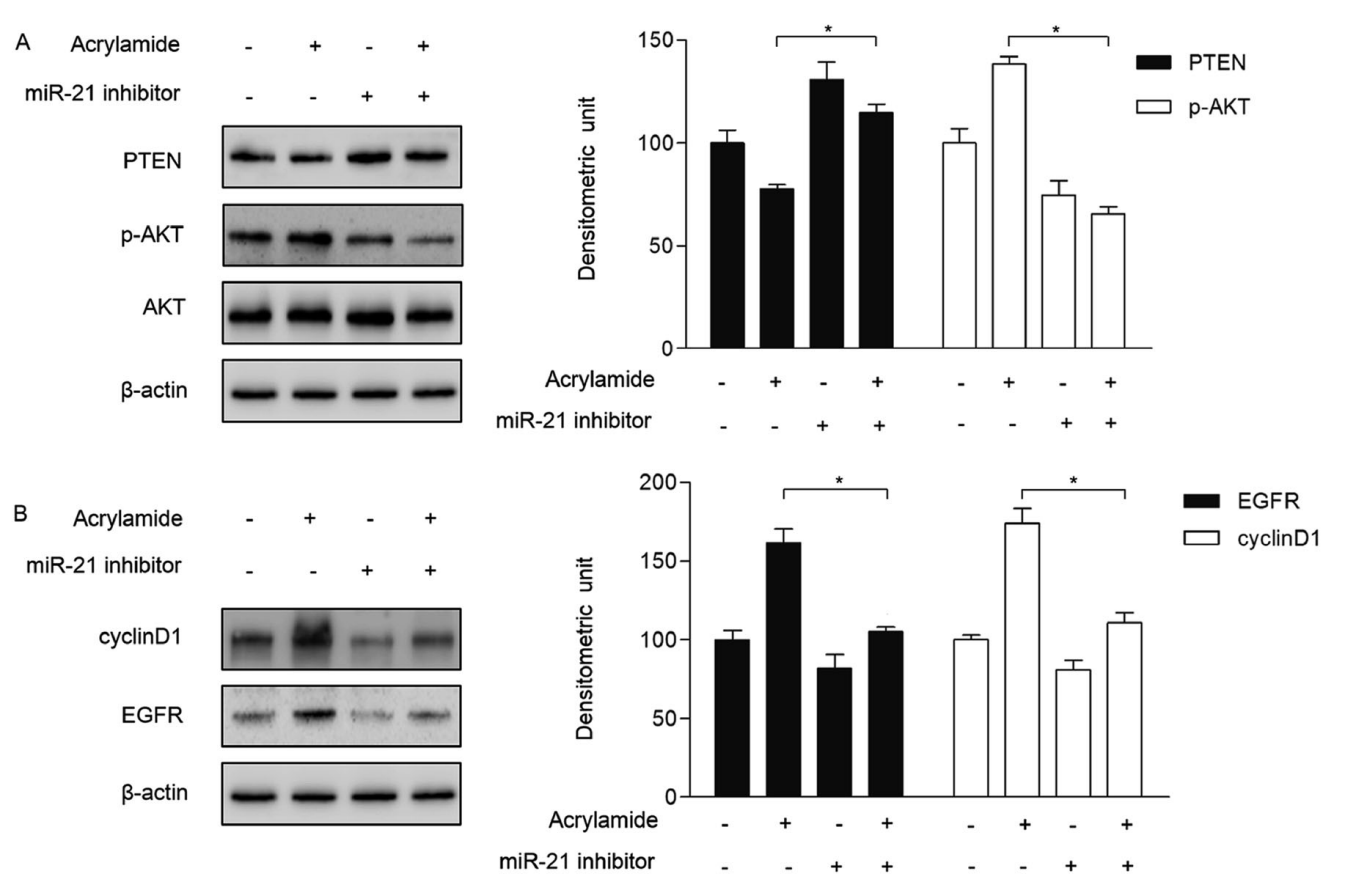
MiR-21 regulated the signaling pathway of miR-21/ PTEN/ AKT in HepG2 cells. A and B: The expression of PTEN, p-AKT, EGFR and cyclin D1 after cells were transfected with miR-21 inhibitor (100 nmol/L) and treated with acrylamide (100 ¦̭ol/L), as determined by Western blotting. *P < 0.05, **P < 0.01.

We have detected that HepG2 cell proliferation was restrained when the cells were transfected with miR-21 inhibitor. Besides, as shown in *Fig. 4A*, miR-21 inhibitor upregulated PTEN expression in HepG2 cells, followed by a decrease of AKT phosphorylation, which downregulated downstream cyclin D1 and EGFR expression (*Fig. 4B*). In the meanwhile, the expression of PTEN was inhibited by acrylamide at 100 µmol/L. Along this line, the levels of AKT phosphorylation, cyclin D1 and EGFR were upregulated (*Fig. 4A* and *B*). In the combination group, the effects of 100 µmol/L acrylamide on p-AKT, cyclinD1 and EGFR were significantly suppressed (*Fig. 4A* and *B*). These results indicated that acrylamide promoted HepG2 cell proliferation through regulating the levels of PTEN and AKT by increasing the expression of miR-21.

### Curcumin suppressed the upregulation of miR-21 in HepG2 cells induced by acrylamide and induced cell apoptosis

**Fig.5:**
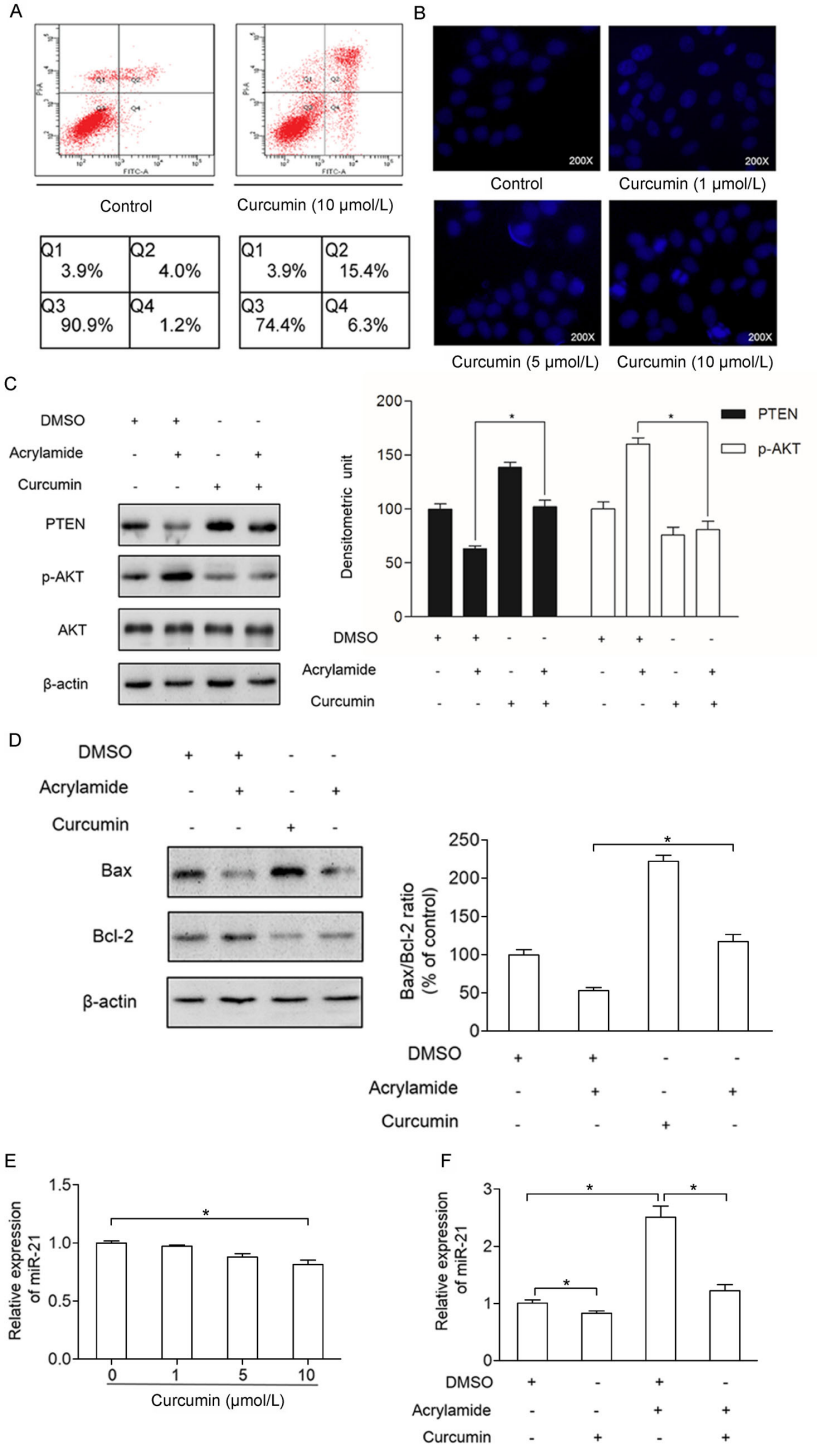
Curcumin suppressed the upregulation of miR-21 in HepG2 cells induced by acrylamide and induced cell apoptosis. A: Apoptosis rate of HepG2 cells after treatment with curcumin (10 ¦̭ol/L) for 24 hours, as measured by flow cytometry analysis. B: HepG2 cell apoptosis after treatment with curcumin (0 ¦̭ol/L, 1 ¦̭ol/L, 5 ¦̭ol/L, 10 ¦̭ol/L) for 24 hours, as assessed by Hoechest 33258 staining. C and D: The level of PTEN, p-AKT, Bcl2 and Bax in HepG2 cells after pre-treatment with curcumin (10 ¦̭ol/L) for 2 hours and combination with acrylamide for 24 hours, as analyzed by western blotting. E: The expression of miR-21 in HepG2 cells after treatment with curcumin (0 ¦̭ol/L, 1 ¦̭ol/L, 5 ¦̭ol/L, 10 ¦̭ol/L) for 24 hours, as detected by qRT-PCR. F: The expression of miR-21 in HepG2 cells, after pre-treatment by curcumin (10 ¦̭ol/L) for 2 hours and combination with acrylamide for 24 hours, as detected by qRT-PCR. *P < 0.05, **P < 0.01.

Our previous study found that curcumin can lower HepG2 cells viability^[19]^. To test whether it can cause cell apoptosis, annexin-V/PI staining and Hoechst 33258 were performed. As shown in *Fig. 5A*, the apoptosis rate of HepG2 cells was increased when the cells were treated with curcumin (10 µmol/L), which was tested by annexin-V/PI staining. Also, Hoechst 33258 indicated that the apoptosis rate of HepG2 cells was increased as the concentrations of curcumin went up (*Fig. 5B*). To further detect the inhibitory effect of curcumin on acrylamide-induced HepG2 cell proliferation, the critical proliferation and apoptosis factors, such as PTEN, p-AKT, Bcl2 and Bax were explored. As shown in *Fig. 5C *and *D*, the expressions of PTEN and Bax were increased significantly in the group with curcumin (10 µmol/L) pretreatment for 2 hours compared to those in the control group. In contrast, the expressions of p-AKT and Bcl2 has decreased in the group pretreated with curcumin (10 µmol/L) for 2 hours compared to those in the control group. Also, the level of PTEN, p-AKT, Bcl2, Bax and Bax/Bcl2 ratio in combination group compared to the acrylamide treatment group showed the inhibitory effect of curcumin on acrylamide inducing HepG2 cell proliferation. What's more, the expression of miR-21 was reduced in a dose-dependent manner when the HepG2 cells were treated with curcumin in various concentrations (0 µmol/L, 1 µmol/L, 5 µmol/L, 10 µmol/L) for 24 hours (*Fig. 5E*). As shown in *Fig. 5F*, acrylamide induced the expression of miR-21, which can be reversed by curcumin.

### Mutual regulation of CYP2E1 and miR-21

**Fig.6:**
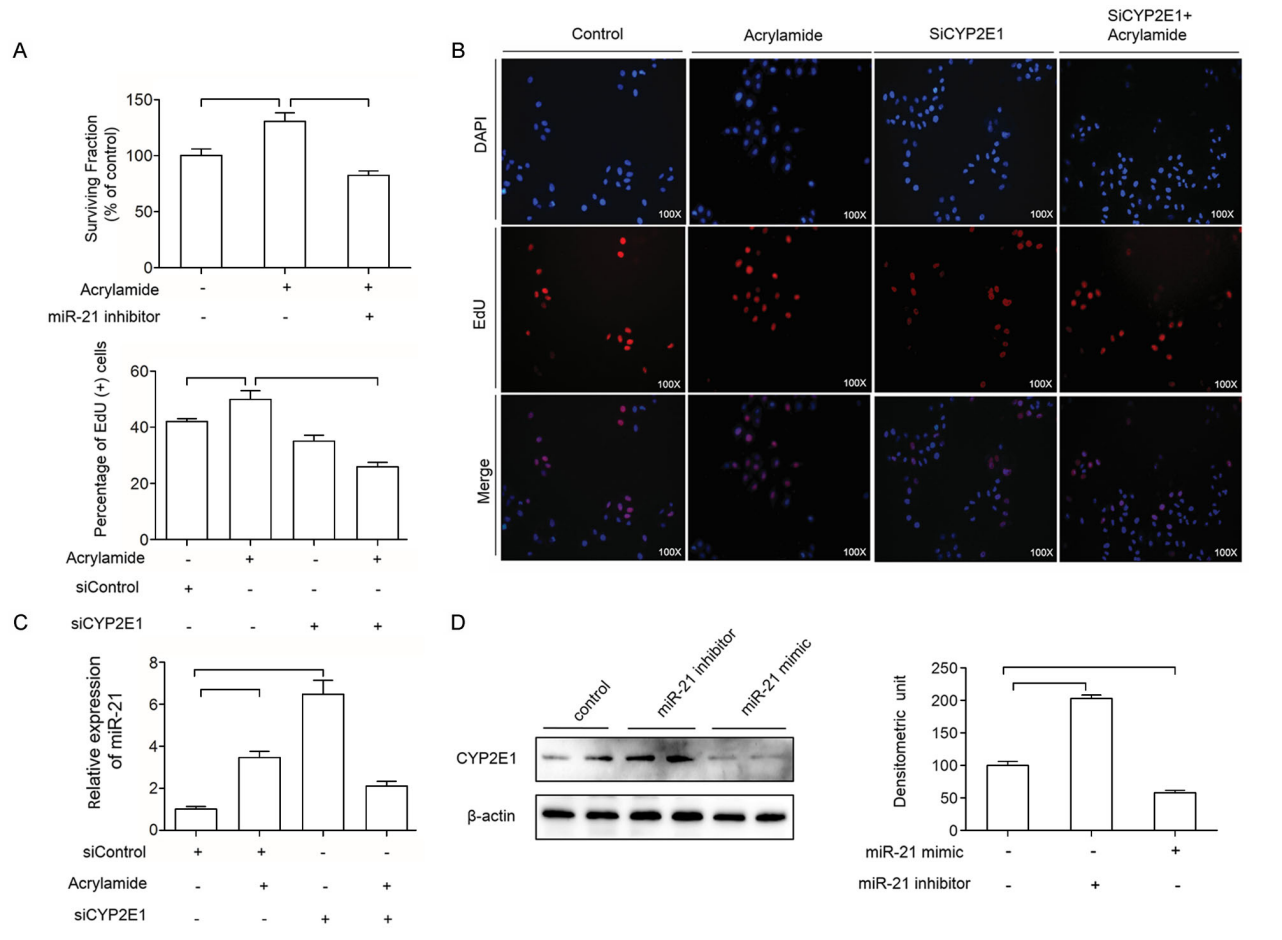
Mutual regulation of CYP2E1 and miR-21. A and B: The cell viability of HepG2 cells after the treatment with acrylamide (100 ¦̭ol/L) alone, combination with miR-21 inhibitor (100 nmol/L), siCYP2E1 alone and combination with siCYP2E1 for 24 hours, as measured by MTT assay and EdU staining. C: The expression of miR-21 after the cells were treated with acrylamide (100 ¦̭ol/L), siCYP2E1, and the combination of acrylamide and siCYP2E1 for 24 hours, as detected by qRT-PCR. D: The expression of CYP2E1 in HepG2 cells after transfection with miR-21 inhibitor (100 nmol/L) or miR-21 mimic (20 nmol/L) for 24 hours, as measured by Western blotting. *P < 0.05, **P < 0.01.

Our previous investigation has verified that acrylamide can induce HepG2 cell proliferation through elevating CYP2E1^[19]^. To investigate the effect of CYP2E1 and miR-21 on cell proliferation, the expression of CYP2E1 and miR-21 was inhibited by siRNA targeting CYP2E1, miR-21 inhibitor. The cell viability of HepG2 was decreased when the cells were treated with miR-21 inhibitor (*Fig. 6A*). Also, the rate of proliferation of HepG2 cells was decreased when the cells were transfected with siCYP2E1 and combination with acrylamide compared to that in the acrylamide group (*Fig. 6B*). Therefore, miR-21 inhibitor and siCYP2E1 can reverse the HepG2 cell proliferation induced by acrylamide. To explore the regulatory relationship between CYP2E1 and miR-21 in HepG2 cells, miR-21 level was testified. After knockdown of CYP2E1 by transfection of siRNA targeting CYP2E1, miR-21 expression was elevated shown in *Fig. 6C*. Also, to further determine the effect of miR-21 on CYP2E1, miR-21 inhibitor and miR-21 mimic were transfected into HepG2 cells. The results displayed that miR-21 inhibitor could increase the level of CYP2E1 meanwhile miR-21 mimic decreased CYP2E1 expression to a certain extent (*Fig. 6D*). All the results above showed that the regulation between miR-21 and CYP2E1 achieved a balance, and promoted cell proliferation together after HepG2 cells were treated with acrylamide.

## Discussion

Studies have indicated that miRNAs exert important influences on tumorigenesis and prevention of cancer through degrading the target mRNA. Also, aberrant expression of miRNA is associated with many types of cancer^[11]^. Although the overexpression of miR-21 is associated with hepatocellular carcinoma cell proliferation^[13]^, few studies reported that acrylamide could produce effects of mediation through miRNAs. In this study, we observed that the acrylamide induced both HepG2 cell proliferation and miR-21 expression in time- and dose-dependent manners for the first time. Hence, we speculated that miR-21 may be involved in the process of HepG2 cell proliferation induced by acrylamide.

It has been verified that miR-21 overexpression is able to induce cell proliferation and carcinogenesis *via* PTEN/PI3K/AKT pathways^[20^–^21]^. PTEN, the target of miR-21, is downregulated by acrylamide in our study. Additionally, PI3K/AKT signaling pathway, the downstream of PTEN, plays proliferative promotion and anti-apoptotic roles *via* mediating the downstream target genes, such as mTOR, NF-κB, and EGFR, which are activated in cancers^[22^–^24]^. In our study, we found that acrylamide increased p-AKT expression, which mediated the overexpression of proliferation-related gene proteins EGFR and cyclin D1. Moreover, LY294002, the inhibitor of PI3K/AKT suppressed the AKT phosphorylation and downregulated its downstream proteins. Then, transfection of miR-21 inhibitor to HepG2 cells, increased the expression of PTEN and decreased the expression of p-AKT as well as the downstream related gene expression. Taken together, acrylamide can inhibit the expression of PTEN, activate AKT phosphorylation and promote cell proliferation in HepG2 cells. Moreover, in this study, Bcl2 expression was upregulated by acrylamide while Bax expression was downregulated. Combination with curcumin can reverse the effect of acrylamide, which was consistent with the anti- or pro-apoptotic effect of Bcl2 family.

CYP2E1, an inducer of oxygen free radicals, has been reported to play a critical role in DNA damage induced by oxidative stress, and it is related to hepatocarcinogenesis associated with ethanol^[25]^. Likewise, CYP2E1 induction exerts important influences on activation of pro-carcinogens and the progressive hepatic disease^[26]^. Our results indicated that acrylamide increased overexpression of CYP2E1 and miR-21. Meanwhile, a certain mechanism of mutual regulation may exist between the miR-21 and CYP2E1. It is the first time that we have associated miR-21 with CYP2E1 in HepG2 cells. Hepatocyte nuclear factor 4-alpha (HNF4α) that plays functional roles in liver development is at a relatively lower level in metastatic hepatocellular carcinoma. In addition, miR-21 can reduce the expression of HNF4α *via* inhibiting the HNF4α-3′UTR activity^[27]^. What's more, HNF4α also mediated the expression of CYP2E1 in HepG2 cells^[28]^. In our study, miR-21 downregulated the expression of CYP2E1, possibly through the transcription factor HNF4α that remains to be further elucidated.

Acrylamide has been previously reported to determine the carcinogenicity in rodents *via* its transforming to glycidamide, though both acrylamide and glycidamide are able to generate DNA damage and cytotoxicity^[7^,^29]^. Nevertheless, it remains uncertain that carcinogenicity can be induced by acrylamide in humans. Exposure of acrylamide in European adults is estimated to vary from 0.3 to 1.1 μg/kg bw/d, according to the contents of acrylamide in food^[30]^. Also, the doses of acrylamide in animal experiments are higher than daily human exposure. In vitro, HepG2 cells were treated with 1.25 mMor 2.5 mmol/L acrylamide, which increased the expression of CYP2E1 and glutathione S-transferase (GST) that in turn elevated the level of carcinogenicity^[31]^. In our previous studies, the rate of cell proliferation and the expression of miR-21 reached the highest level when HepG2 cells were treated with 100 µmol/L acrylamide^[19]^. Therefore, we speculate on the molecular mechanism of acrylamide at 100 μmol/L in HepG2 cell proliferation. Besides, curcumin can reverse the proliferation-promoting effect of acrylamide even at the concentration of 100 μmol/L^[19]^.

Large numbers of investigations showed that curcumin exerts anticancer and anti-inflammatory effects by many pathways^[32^–^33]^. In addition, numerous reports showed that curcumin is involved in the regulation of a few microRNAs such as miR-192, Let-7a, and miR-21^[21^,^34]^. Previous researches indicated that curcumin antagonizes the increased level of ROS induced by acrylamide^[35]^. In this study, we observed that curcumin at the concentration of 10 µmol/L can inhibit the upregulation of miR-21 induced by acrylamide, though the systemic bioavailability of curcumin is low. Studies supported the application of curcumin as an anti-cancer drug^[36^–^37]^. However, the low bioavailability of curcumin is a challenge* in vitro *and *in vivo*^[38]^. Therefore, it is imperative to develop new and comprehensive methods for increasing the bioavailability of curcumin. Furthermore, to improve this study, we will try to use primary hepatocytes and more than 2 cell lines (QGY-7703 and SMMC-7721) and animal model will be added in the further study.

In conclusion, the results in this study indicated that natural polyphenol curcumin can reverse the proliferation-promoting effect of acrylamide and induce apoptosis in HepG2 cells, in which miR-21 is involved. The data provided a potential target of cancer prevention and treatment.
